# Regulatory Effects of Histone Deacetylase Inhibitors on Myeloid-Derived Suppressor Cells

**DOI:** 10.3389/fimmu.2021.690207

**Published:** 2021-06-02

**Authors:** Yudan Cui, Jingshan Cai, Wenxin Wang, Shengjun Wang

**Affiliations:** ^1^ Department of Laboratory Medicine, The Affiliated People’s Hospital, Jiangsu University, Zhenjiang, China; ^2^ Department of Immunology, Jiangsu Key Laboratory of Laboratory Medicine, School of Medicine, Jiangsu University, Zhenjiang, China

**Keywords:** acetylation, histone deacetylases, inhibitors, myeloid-derived suppressor cells, immunosuppression

## Abstract

Histone deacetylase inhibitors (HDACIs) are antitumor drugs that are being developed for use in clinical settings. HDACIs enhance histone or nonhistone acetylation and promote gene transcription *via* epigenetic regulation. Importantly, these drugs have cytotoxic or cytostatic properties and can directly inhibit tumor cells. However, how HDACIs regulate immunocytes in the tumor microenvironment, such as myeloid-derived suppressor cells (MDSCs), has yet to be elucidated. In this review, we summarize the effects of different HDACIs on the immunosuppressive function and expansion of MDSCs based on the findings of relevant studies.

## Introduction

Myeloid-derived suppressor cells (MDSCs) are heterogeneous cells derived from bone marrow that can suppress the immune response (1, 2). MDSCs are produced in large quantities under pathological conditions, such as inflammation and cancer. The accumulation of MDSCs is a complex and gradual phenomenon that is regulated by many factors (3). MDSCs are composed of two major types of cells: the granulocytic or polymorphonuclear type (PMN-MDSCs), which are similar to neutrophils in phenotype and morphology, and the monocytic type (M-MDSCs), which are similar to monocytes in phenotype and morphology. In most types of cancer, PMN-MDSCs account for more than 80% of all MDSCs, while M-MDSCs are direct promoters of tumor metastasis (4). In mice, MDSCs are more common in tumors of the bone marrow, spleen, liver and other organs. PMN-MDSCs and M-MDSCs are defined as CD11b^+^Ly6G^+^Ly6C^lo^ and CD11b^+^Ly6G^-^Ly6C^hi^, respectively. In humans, MDSCs are most common in the blood and tumors of various organs. In peripheral blood mononuclear cells, PMN-MDSCs are defined as CD11b^+^CD14^-^CD15^+^ or CD11b^+^CD14^-^CD66b^+^, while M-MDSCs are defined as CD11b^+^CD14^+^HLA-DR^-/lo^CD15^-^. Lin^-^HLA-DR^-^CD33^+^ cells are a group of mixed MDSCs containing more immature progenitor cells that have been proposed to be defined by ‘early-stage MDSCs’ (e-MDSCs). However, the same type of cells have yet to be identified in mice (5). The signals driving the development of MDSCs occur in two partially overlapping stages (6). In the first stage, the expansion and regulation of bone marrow cells occur in the bone marrow and spleen, while the second stage is characterized by the transformation of neutrophils and monocytes into pathologically activated MDSCs, which primarily occurs in peripheral tissues (7). Several factors participate in MDSC-mediated immunosuppression, including arginase-1 (Arg-1), inducible nitric oxide synthase 2 (iNOS), transforming growth factor β (TGF-β), interleukin-10 (IL-10), cyclooxygenase 2 (COX2) and indoleamine 2,3-dioxygenase (IDO) (8, 9). Although MDSCs are involved in the suppression of different cells in the immune system, T cells are the primary targets of MDSCs. Both PMN-MDSCs and M-MDSCs can reduce the production of L-arginine through the expression of Arg-1 and iNOS, thereby inhibiting the function of T cells (10, 11). Furthermore, M-MDSCs and PMN-MDSCs also take advantage of different immunosuppressive mechanisms. M-MDSCs use NO and produce related cytokines to inhibit the ability of T cells to eliminate antigens (12), while PMN-MDSCs primarily inhibit the immune response in an antigen-specific manner. The induction of antigen-specific T cell tolerance is one of the primary characteristics of PMN-MDSCs (13, 14), and reactive oxygen species (ROS) production is crucial for this activity (8). In recent years, an understanding of the clinical importance of MDSCs has emerged. An initial study monitored MDSCs from cancer patients and analyzed the total MDSC population. The results showed that the number of peripheral blood MDSCs was positively correlated with the tumor stage and tumor burden of colorectal, breast, thyroid and nonsmall cell lung cancers (3, 15–21). In melanoma and hepatocellular carcinoma, both PMN-MDSCs and M-MDSCs were shown to be associated with a poorer prognosis (3, 22, 23). In nonsolid tumors, M-MDSC numbers were associated with reduced survival in multiple myeloma, Hodgkin’s lymphoma, non-Hodgkin’s lymphoma, and diffuse large B-cell lymphoma (24–26). Therefore, therapeutics targeting MDSCs have become an important means of tumor immunotherapy by inhibiting their differentiation, expansion and activity.

Immune checkpoint inhibitors, such as anti-PD-1 and anti-CTLA-4, have shown success in eradicating cancer by enhancing immune activation, but primary and secondary resistance are still problems (27). Epigenetic treatments for cancer include histone deacetylase inhibitors (HDACIs), DNA methyltransferase inhibitors (DNMTIs) and histone methyltransferase inhibitors (HMTIs), which can stimulate tumor cells and improve the antitumor response by host immune cells. Epigenetic treatments can improve the response of tumor patients to immune checkpoint blockade therapy (28). DNMTIs have been reported to be effective in the treatment of hematological malignancies in clinical studies (29), while HMTIs have been shown to play a role in the treatment of multiple myeloma (30). However, some DMNTIs and HMTIs have not shown clinical efficacy. HDACIs are a different class of small molecule drugs that can have a wide range of effects on tumor cells, including cell cycle arrest, apoptosis, cell differentiation, autophagy and antiangiogenesis (31). HDACIs can inhibit HDACs, and because these drugs have a more pronounced effect on the proliferation of malignant cells than nonmalignant cells, there is increasing interest in developing these drugs, especially as antitumor treatments. In recent studies, many researchers have found that HDACIs also have significant effects on host immunosuppressive cells. As MDSCs are important immunosuppressive cells in the tumor microenvironment (32), it is worth investigating the regulatory effects of HDACIs these cells.

## Acetylation

Lysine acetylation is an evolutionarily conserved posttranslational modification that occurs in prokaryotes and eukaryotes. In general, two different types of protein acetylation occur in cells. In humans, 80-90% of proteins undergo cotranslational acetylation at the Nα end of the nascent polypeptide chain (43–45). The other common type of protein acetylation occurs at the ϵ-amino group of lysine. Acetylation was first discovered in histones (46). Subsequently, researchers observed acetylation modifications on nonhistones and identified histone acetyltransferases (HATs) and histone deacetylases (HDACs). In the past decade, advances in proteomics based on mass spectrometry have greatly expanded the classification of endogenous acetylated proteins, provided an objective perspective for the study of acetylation, and provided new insights into the scope and regulation of nonhistone acetylation. To reflect the degree of nonhistone acetylation, HATs and HDACs were renamed lysine acetyltransferases (KATs) and lysine deacetylases (KDACs), respectively (47) (ordinarily, the terms HATs and HDACs are used). Acetylation is a dynamic and reversible process involving both KATs and KDACs. KATs are responsible for covalently attaching an acetyl group to the lysine residue of a protein and are figuratively called “writers”, while KDACs mediate the removal of this acetyl group and are called “erasers”. Acetylation is the addition of acetyl groups to lysine residues in a protein that occurs in the presence of acetyl transferase. Acetylation is an important type of posttranslational modification for acetyl-CoA metabolism and cell signal transduction. In addition, acetylation is a widespread regulatory mechanism mediated by posttranslational modification in the subcellular organelles of the nucleus or cytoplasm and is involved in many processes, such as transcription, chemotaxis, metabolism, cell signal transduction, stress response, proteolysis, cell apoptosis, and neuron development (47). Evidence has shown that acetylation is one of the most important modifications used to alter protein activity and precisely regulate and control cellular functions.

## Histone Deacetylases

HDACs can mediate the deacetylation of histones and nonhistone proteins and are a class of proteases that play important roles in chromosome structural modifications and gene expression regulation (48). Under normal conditions, the acetylation of histones is beneficial for the dissociation of DNA and dense histone octamers, allowing the nucleosome structure to relax so that various transcription factors and cooperative transcription factors can bind to specific DNA binding sites and activate gene transcription. The deacetylation of histones has the opposite effect (49). In addition to regulating histone modification, HDACs also regulate the posttranslational acetylation of many nonhistones, including transcription factors, chaperones, and signaling molecules, leading to changes in protein stability, protein-protein interactions, and protein-DNA interactions (50). There are four classes of HDACs. Class I includes HDAC1, HDAC2, HDAC3 and HDAC8. Class II is further divided into IIa and IIb, with HDAC4, HDAC5, HDAC7 and HDAC9 belonging to class IIa, while class IIb includes HDAC6 and HDAC10. Class III is composed of sirtuin1-7, and class IV includes HDAC11 only. Classes I, II and IV enzymes are zinc ion dependent, while class III members are zinc ion independent (51). It is worth noting that nearly half of all deacetylases have weak or no deacetylase activity or target other types of acylation (47).

## Histone Deacetylase Inhibitors

HDACIs can inhibit the deacetylation of histones or nonhistone proteins and have direct inhibitory effects on tumor cells. Inhibiting HDACs can regulate the balance between proapoptotic and antiapoptotic proteins, leading to the death of tumor cells (52). While HDACIs have direct inhibitory effects on tumor cells, they can also regulate various components of the host immune system (53). Some researchers have found that the treatment of cancer patients with HDACIs can reduce the number of lymphocytes, indicating that HDACIs are immunocytotoxic (54, 55). On the other hand, some researchers have shown that HDACIs promote immune activity and can enhance cancer immunotherapy (56–58). Theoretically, targeted inhibition of HDACs is closely associated with adverse outcomes after trauma and can optimize treatment outcomes while reducing complications (59). Many isotype-specific HDACIs are now available and are undergoing clinical trials as antitumor agents (60). HDACIs can be structurally classified into at least four categories (hydrochlorates, cyclic peptides, fatty acids, and benzoamides) and can also be classified according to their HDAC specificity. Broad-spectrum HDACIs include panobinostat, belinostat, resminostat and trichostatin A. Butyrate and valproate inhibit class I and IIa HDACs. Romidepsin, entinostat (ENT) and mocetinostat are considered class I specific, and tubacin is HDAC6 specific (51). Due to the zinc ion-dependent nature of the domains of class I, II and IV HDACs, inhibitors occupying the zinc ion-binding site of the catalytic center will inhibit the activity of these enzymes. These HDACIs contain a pharmacophore, a cap structure, a linking unit and a zinc ion-binding group to chelate cations in the catalytic region of the target HDACs (27). Trichostatin A, vorinostat, belinostat, dacinostat, panobinostat and givinostat are HDACIs. Recent studies have shown that HDACIs also have crucial effects on host immunosuppressive cells, with MDSCs being important immunosuppressive cells in the tumor microenvironment (**Table 1**).

**Table 1:** Effects of HDACIs on MDSCs.

HDACI	Classification	Effects on MDSCs	Ref
Entinostat	Class I	PMN-MDSC function inhibited	(33)
		M-MDSC migration inhibited	(34)
Valproic acid	Class I	PMN-MDSC function inhibited	(35)
		M-MDSC migration inhibited	(36)
Mocetinostat	Class I/IV	total number of MDSCs decreased	(37)
Vorinostat	Class I/II	MDSC apoptosis (at higher vorinostat concentrations)	(38)
		total number of MDSCs amplified (at lower vorinostat concentrations)	(39)
CG-745	Class I/IIb	total number of MDSCs decreased	(40)
ACY241	specific inhibitor of HDAC6	total number of MDSCs decreased	(41)
Trichostatin A	Broad spectrum (except HDAC8)	total number of MDSCs amplified (0.1-10 nM TSA)	(39)
		PMN-MDSC number decreased	(42)

## Effects of HDACIs on MDSCs

### Entinostat

ENT is a specific inhibitor of class I HDACs that targets immunosuppressive cells in the tumor microenvironment (61). ENT has been reported to have immunoregulatory activity (62) and has been used in the clinical treatment of breast and nonsmall cell lung cancers (63, 64). The clinical drug development of ENT focuses on the resistance mechanism of breast cancer to endocrine therapy and HER2-targeted drugs (63). Importantly, ENT can inhibit tumor cell proliferation, which can induce mitochondrial damage and lead to apoptosis. ENT increases the sensitivity of lung cancer cells to tumor necrosis factor-related apoptosis-inducing ligands and downregulates the expression of the antiapoptotic genes Bcl-2 and XIAP (64).

**Figure 1:**
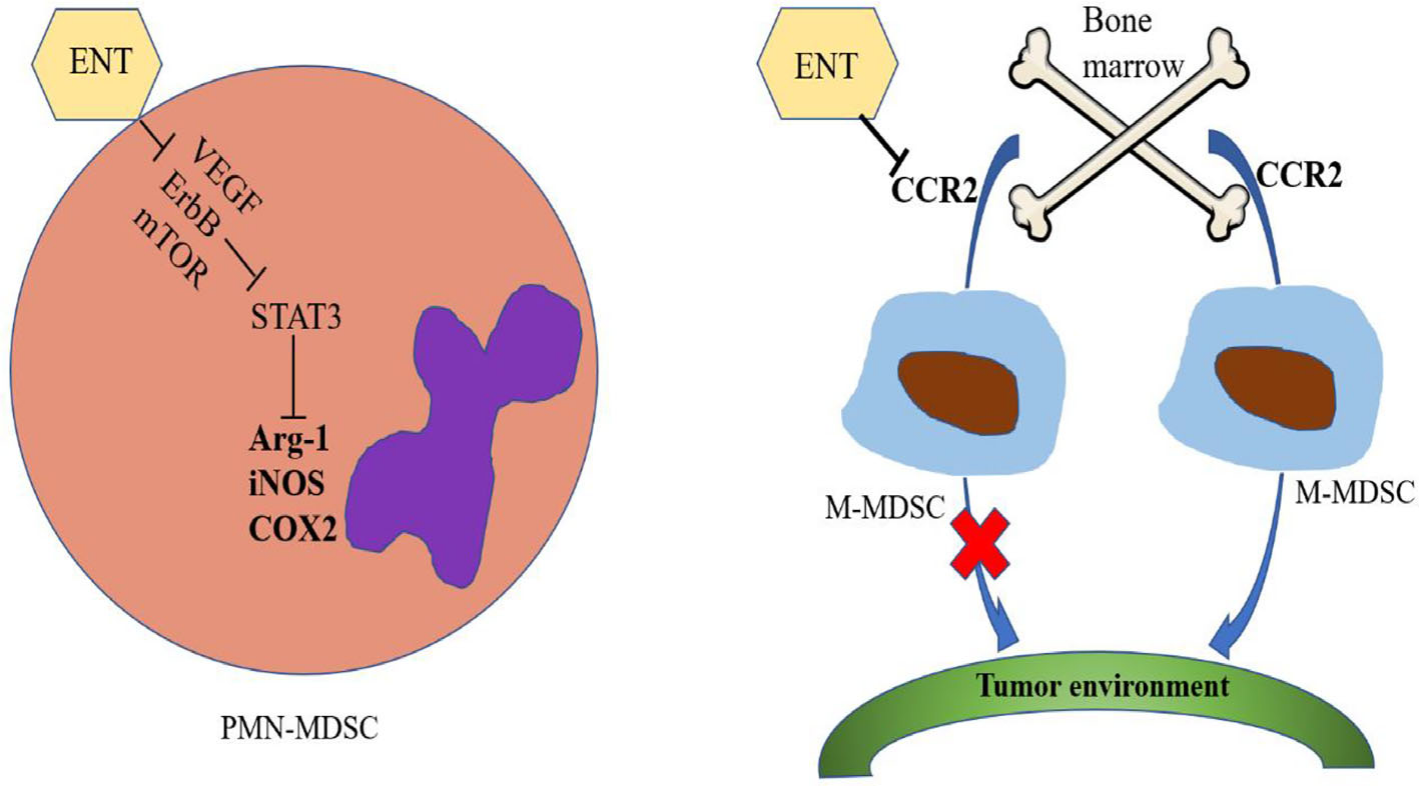
Effects of entinostat on MDSCs. Entinostat inhibits the VEGF, ErbB and mTOR pathways in PMN-MDSCs, thereby inhibiting the activity of STAT3, which in turn reduces the activities of Arg-1, iNOS and COX2. Entinostat therapy inhibits the transport of M-MDSCs from bone marrow to the tumor environment by downregulating CCR2 expression.

Using lung and renal cell carcinoma models, Orillion A and colleagues observed that the total number of MDSCs in tumors increased in the presence of ENT alone but only slightly increased after treatment with ENT combined with anti-PD-1. In addition, there was also a decline in immunosuppressive functions, showing that ENT can inhibit the levels of Arg-1, iNOS and COX2, thereby reducing the immunosuppressive effects of MDSCs (62). After treatment with ENT, the tumor-free survival of HER/neu transgenic breast cancer and Panco2 metastatic pancreatic cancer mouse models was significantly improved. ENT combined treatment with anti-PD-1 and anti-CTLA-4 was shown to inhibit the VEGF, ErbB and mTOR pathways in PMN-MDSCs as well as the activity of STAT3 and the activity of Arg-1 (33). Tomita et al. reported that the number of circulating PMN-MDSCs and M-MDSCs decreased in samples from breast cancer patients treated with ENT combined with an aromatase inhibitor (65). However, the immunosuppressive activity of PMN-MDSCs could specifically be reduced by ENT treatment, and there was no effect on M-MDSCs (66). The microenvironment before tumor metastasis has been shown to be established through the activities of M-MDSCs, suggesting that the number of M-MDSCs and niche-promoting molecules in the lung tissue before tumor metastasis can be reduced by low-dose 5-azacytidine (100 nM) and low-dose ENT (50 nM) treatment. Interestingly, the gene set related to the chemokine axis and immune cell migration was observed to be significantly altered by low-dose ENT treatment, and the expression of CCR2 in M-MDSCs in the bone marrow and lung was significantly downregulated after low-dose ENT treatment (34). CCR2 is a key regulator of the migration of M-MDSCs from the bone marrow to the tumor environment, suggesting that the transport of M-MDSCs to the premetastatic lung may be affected by low-dose ENT therapy at least partially through the downregulation of CCR2 (67, 68). In short, ENT can inhibit the function of PMN-MDSCs and the metastasis of M-MDSCs (**Figure 1**).

### Valproic Acid

Valproic acid (VPA) is an anticonvulsant drug (69) and an HDACI (70, 71) that targets HDAC class I enzymes (HDAC1, 2 and 3). *In vitro* experiments by Xie Z et al. showed that VPA treatment can reduce the proportion of PMN-MDSCs, inhibit the immunosuppressive function of MDSCs in a dose-dependent manner and also reduce the level of Arg-1 by inhibiting IL-4Rα expression, thereby weakening the immunosuppressive function of MDSCs (35). In a recent study, VPA was shown to downregulate CCR2 expression in M-MDSCs, and the tumor invasion ability of these cells was also reduced (36). In addition, VPA was shown to decrease the immunosuppressive effects of MDSCs on CD8^+^ T cells and NK cells, and the ability of these cells to kill tumors was also enhanced (36). Moreover, treatment with VPA combined with an anti-PD-L1 antibody blocked the immunosuppressive functions of MDSCs by activating IRF1/IRF8 (72).

### Mocetinostat

Mocetinostat is a selective inhibitor of class I/IV HDAC, proteins involved in the epigenetic silencing of immunoregulatory genes in tumors and immune cells. The target gene promoters of mocetinostat are occupied by class I HDACs, and an increase in active histone markers is observed after mocetinostat treatment (37). Briere D and colleagues suggested that the number of MDSCs and Tregs could be reduced by mocetinostat treatment, with an increase in CD8^+^ T cells observe in a tumor-bearing mouse model of colorectal cancer (37). However, the exact mechanism of action of mocetinostat remains unclear.

### Vorinostat

Vorinostat (SAHA) is a nonspecific inhibitor of class I and class II HDACs and was the first HDACI drug approved by the Food And Drug Administration for clinical use in patients with cutaneous T cell lymphoma (73). Vorinostat can also reduce acute graft-versus-host disease (GVHD) after allogeneic bone marrow transplantation by inhibiting the production of proinflammatory cytokines such as TNF-α, IL-1 and IFN-γ (73).

MDSCs were shown to be induced by both GM-CSF and vorinostat-induced tumor pressure *in vitro*, which can mediate MDSC apoptosis and contradicts the results of other researchers, possibly because different concentrations of vorinostat were used (38). In a spontaneous transgenic mouse melanoma model, treatment with vorinostat resulted in a significant delay in disease onset, downregulation of chemokine (c-c motif) ligand 2 (CCL2) and the recruitment of MDSCs (74). Kroesen M and colleagues showed that the number of M-MDSCs in the tumor microenvironment of 9464D tumor-bearing mice could be reduced by vorinostat treatment. Thus, vorinostat can create a permissible tumor microenvironment for tumor-directed mAb therapy by increasing the number of macrophage effector cells expressing high levels of Fc receptors (75).

### CG-745

CG-745 is a specific inhibitor of class I and class IIb HDACs that exhibits anticancer effects on pancreatic, colorectal and nonsmall cell lung cancers (56). Kim YD and colleagues analyzed the distribution of immune cells in the tumor microenvironment and spleen and reported that CG-745 could inhibit M2 macrophage polarization and reduce the number of MDSCs (40). Therefore, the cytotoxicity of PBMCs and IFN-γ expression in Jurkat T cells could be increased by CG-745. H3 acetylation, which is an important factor during the differentiation of naïve CD8^+^ T cells into memory T cells, was also induced (76).

### ACY241

ACY241 is a specific inhibitor of HDAC6 that inhibits multiple myeloma when used in combination with immunoregulatory drugs and proteasome inhibitors (41). After treatment with ACY241, the number of MDSCs in patients with multiple myeloma was shown to significantly decrease. Bcl6 expression in CD8^+^ T cells may be enhanced by ACY 241 through activation of the AKT/mTOR/NF-κB signaling pathway in CD8^+^ T cells, thereby enhancing CD8^+^ T cell activity (41).

### Trichostatin A

Trichostatin A (TSA) is a natural antifungal metabolite produced by *Streptomyces* and is a broad-spectrum HDACI with no effect on HDAC8 (77). Rosboroug BR et al. observed that after GM-CSF-induced mouse bone marrow cells were treated with TSA (0.1-10 nM) and vorinostat (10-500 nM), CD11b+ GR1+ cells and MDSCs were strongly amplified (39). After TSA treatment of experimental autoimmune encephalomyelitis, PMN-MDSCs were present in reduced numbers in secondary lymphoid organs and migrated into the spinal cord without affecting monocytes, while the disease symptoms improved (42). Additionally, the numbers of Tregs and MDSCs were reduced in Her2/CT26 tumor-bearing mice treated with TSA (78).

## Conclusion

In general, most HDACIs inhibit class I or class II HDACs. Among these molecules, the regulatory effects of entinostat on MDSCs have been reported the most often, probably because entinostat has been put into clinical use. In summary, entinostat inhibits the VEGF, ErbB and mTOR pathways in PMN-MDSCs, thereby inhibiting the activity of STAT3, which in turn reduces the activity of Arg-1, iNOS and COX2. Entinostat also inhibits the transport of M-MDSCs from bone marrow to the tumor environment by downregulating CCR2. Other HDACIs have antitumor effects by reducing the number of MDSCs, but the specific mechanism of action varies. Interestingly, increased concentrations of vorinostat can amplify the number of MDSCs. Why different concentrations of vorinostat lead to different results is worth further research. At the same time, these results suggest that different doses of HDACIs may have different effects, demonstrating that studies of HDACIs must involve strict control of the drug dose. HDACIs have been shown to be effective antitumor agents in clinical studies, but their success has been limited. In addition, these inhibitors can produce side effects, such as platelet reduction, nausea, vomiting, anorexia and fatigue.

In recent years, research on MDSCs has gradually increased, and some researchers regard MDSCs as targets of tumor therapy. Therefore, it is necessary to explore the regulatory effects of HDACIs on MDSCs, which may improve their therapeutic effects toward tumors.

## Author Contributions

YC drafted the manuscript. JC and WW discussed and revised the manuscript. SW designed the study and revised the manuscript. All authors contributed to the article and approved the submitted version.

## Funding

This work was supported by the Research Project of the Jiangsu Commission of Health (grant No. K2019019) and the Jiangsu Province’s Key Medical Talents Program (grant No. ZDRCB2016018).

## Conflict of Interest

The authors declare that the research was conducted in the absence of any commercial or financial relationships that could be construed as a potential conflict of interest.
